# Supplementary material for: The transcriptional legacy of developmental stochasticity
Source: Nat Commun. 2023 Nov 9;14:7226. doi: 10.1038/s41467-023-43024-5 (PMC10632366; doi:10.1038/s41467-023-43024-5)
Supplement: Supplementary file 1 — Supplementary Information [file 41467_2023_43024_MOESM1_ESM.pdf]

Fig. S1

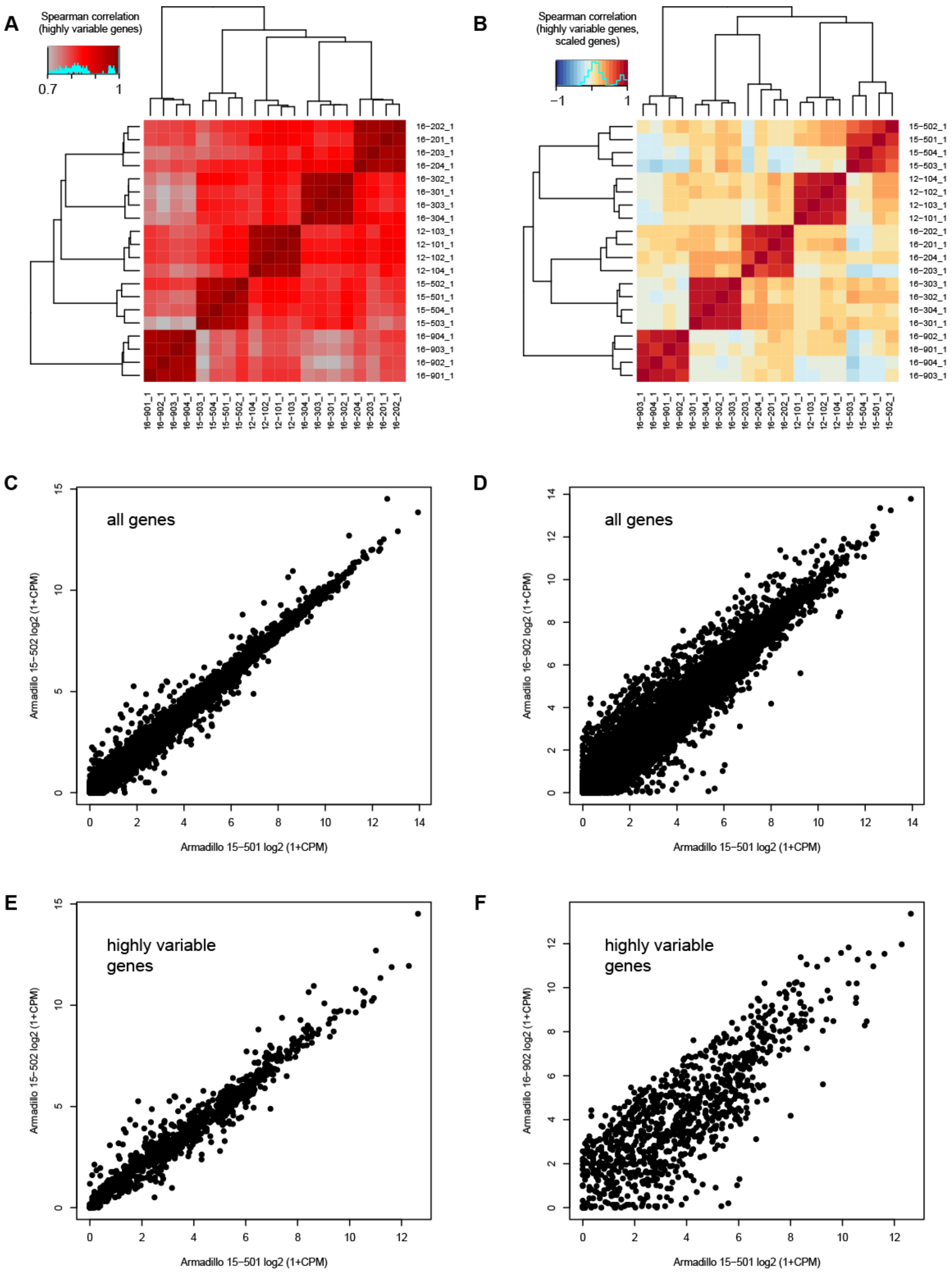

Fig. S1. Transcriptomic similarity of the armadillos. (A) Transcriptional similarity (Spearman correlation) based on top 1,000 highly variable genes. (B) Same as A, except genes are scaled (z-scored) before computing correlations). (C-F) Example scatter plot illustrating transcriptional correlation within (C, E) and across quadruplets (D, F).

Fig. S2

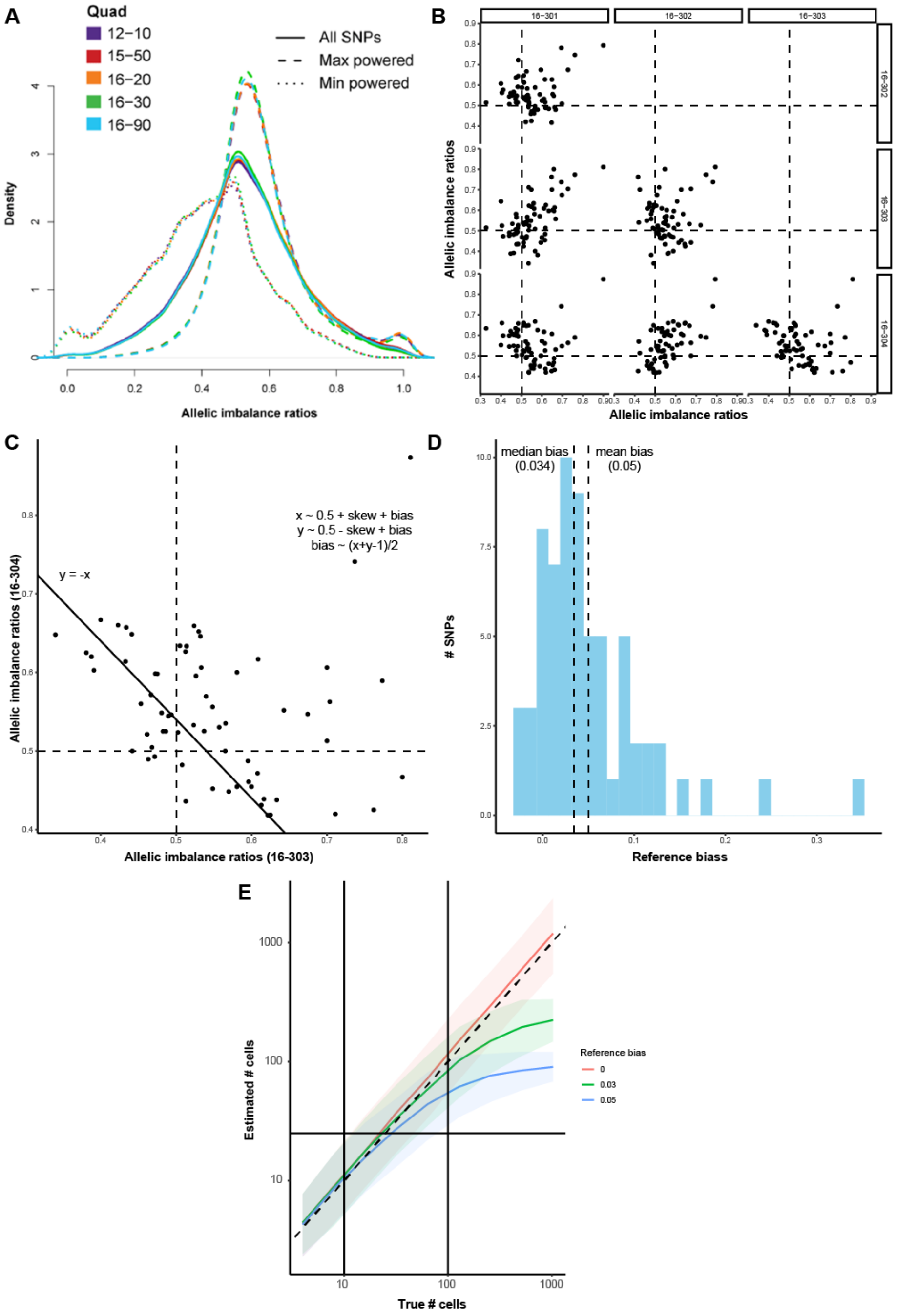

Fig. S2. Impact of reference bias on XCI estimation. (A) Allelic ratios for heterozygous SNPs on the X chromosome show evidence of reference bias (all SNPs, SNPs with most reads per gene, SNPs with fewest reads per gene). (B) Scatter plot showing correlation of allelic ratios for best powered SNPs on the X chromosome for siblings in quad 16-30. Individuals 16-303 and 16-304 have clearly anti-correlated ratios, indicating opposite skews. (C) Focus on allelic ratios of individuals 16-303 and 16-304. Most SNPs fall around the y = -x line, suggesting that these individuals have the same skew, but in opposite direction. The allelic ratio of each SNP can be seen as a combination of skew and reference bias, enabling us to estimate the magnitude of reference bias. (D) Estimation of reference bias for SNPs shown in (C). (E) Simulation-based computation (binomial model) of the impact of reference bias on XCI estimation: an estimated 25 cells is compatible with a true number of cells at XCI ranging from 10 to 100 cells for average reference biases up to 0.05.

Fig. S3

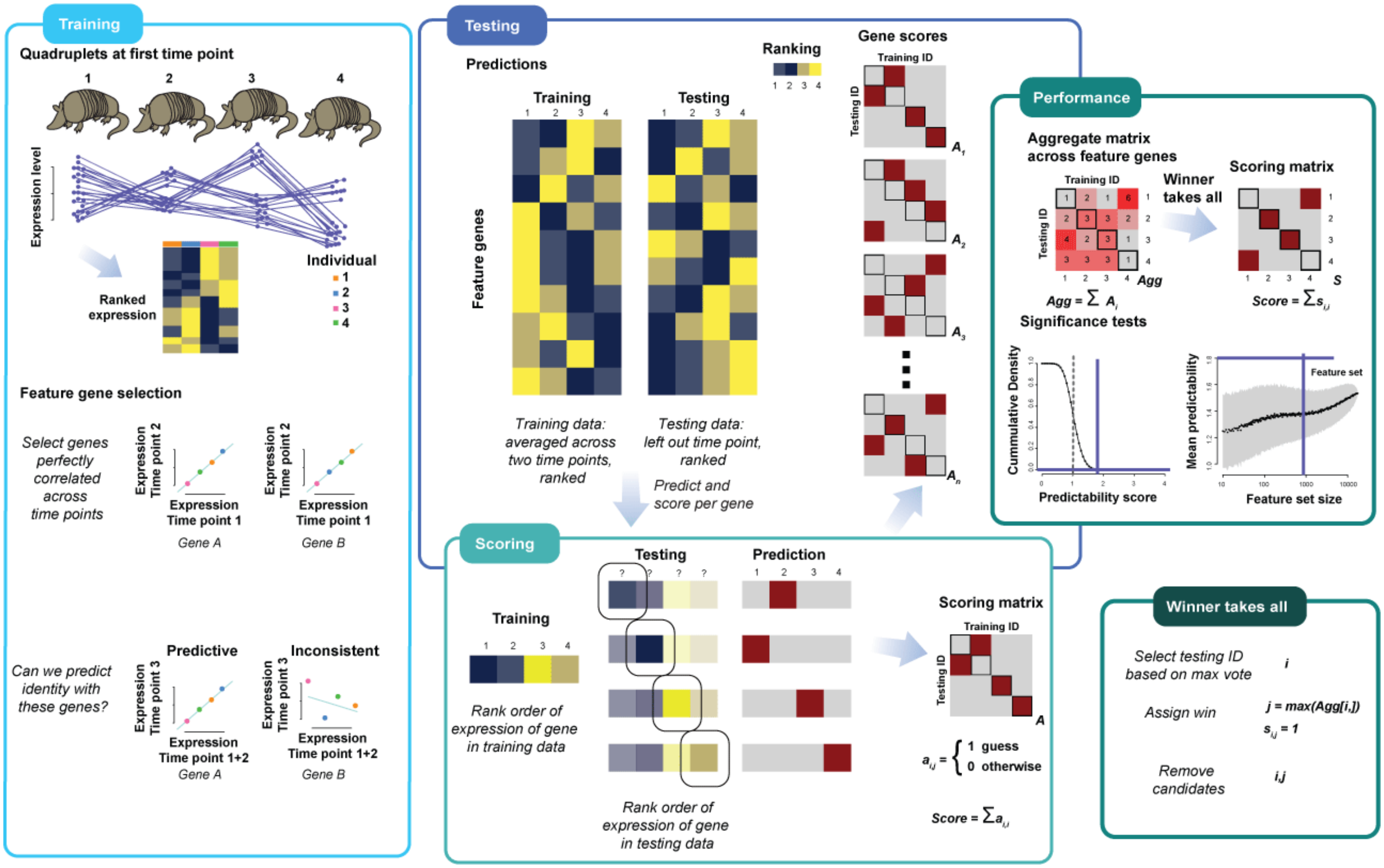

Fig. S3. Testing for individuality. Schematic of machine-learning method. Training: feature set of genes are selected based on correlations between two time points. For each gene, we calculate the Spearman rank correlation between the values across a quadruplet for one time point and a second time point. If the rank ordering is consistent (i.e., the correlation is 1), then this gene is selected as a feature gene. Testing: The first two time points are perfectly correlated, these genes form the training set, and the left out time point is the test set. A gene scoring matrix (4 by 4) is built per gene by comparing the ordering of the test and training data. Each individual gives a score of 1 to the test data individual it thinks it is (i.e., which rank it matches), and a 0 otherwise. Scoring: We then sum all the feature gene scoring matrices to produce an aggregate scoring matrix. Then, in a winner takes all strategy, we calculate a score which represents the number of armadillos that correctly predict themselves. The final score is between 0 and 4, with 4 as perfect predictability i.e., each armadillo correctly identifies its future (or past) self. Performance: We repeat this three times, using the first and second time points as training, the first and third, and finally the second and third, and then testing in the left out time point. We average this across time points to get quadruplet specific scores, and also across all to get a final overall score for the analysis. We calculate an analytic p-value for this score by convolution of the expected distributions. We calculate an empirical p-value by repeating the learning task on randomly selected genes.

Fig. S4

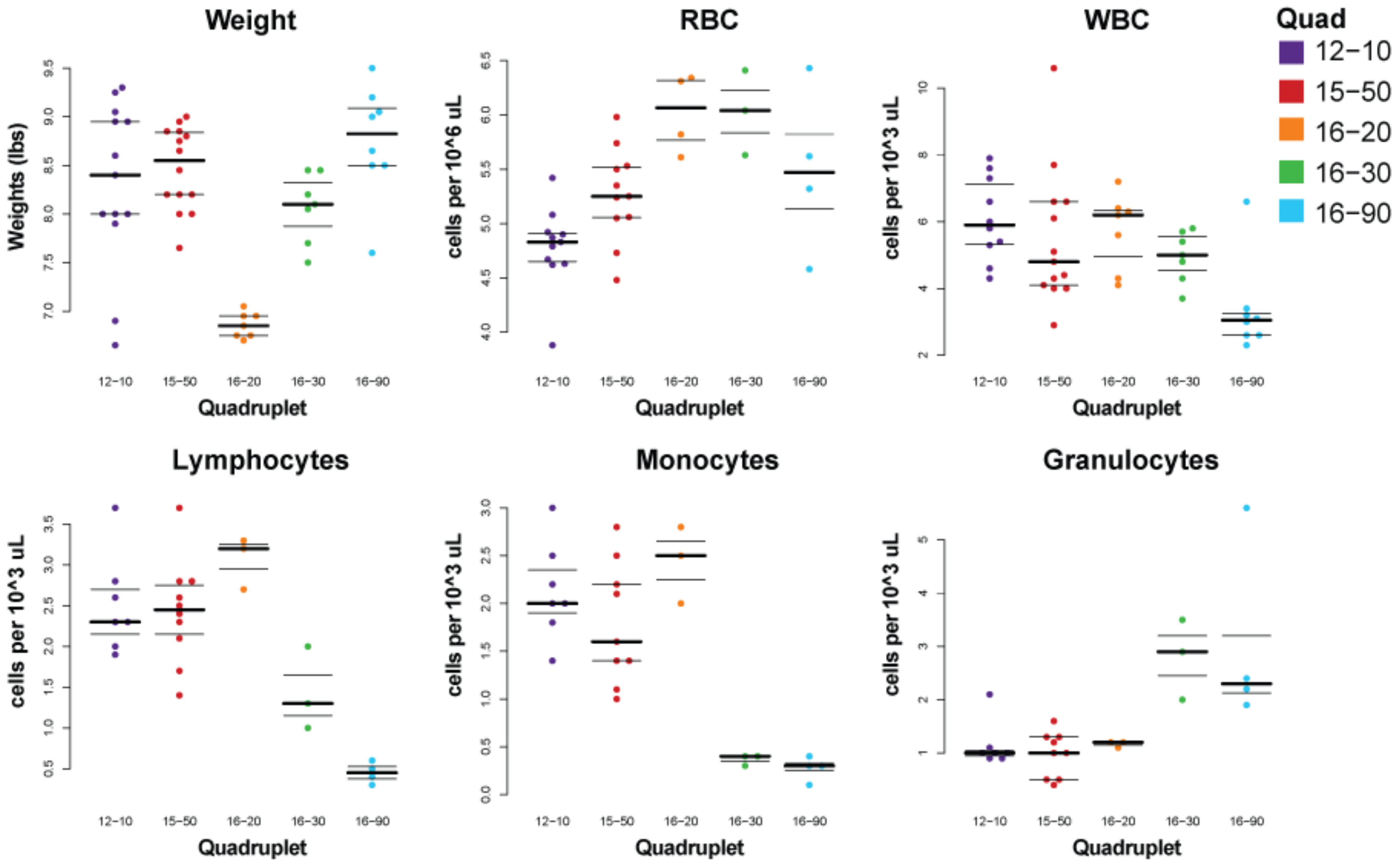

Fig. S4. Weight and blood measurements of the armadillos. Multiple time points are included in the top row, showing consistency across time and within quadruplet. The bottom row shows cell counts for our third time point.

Fig. S5

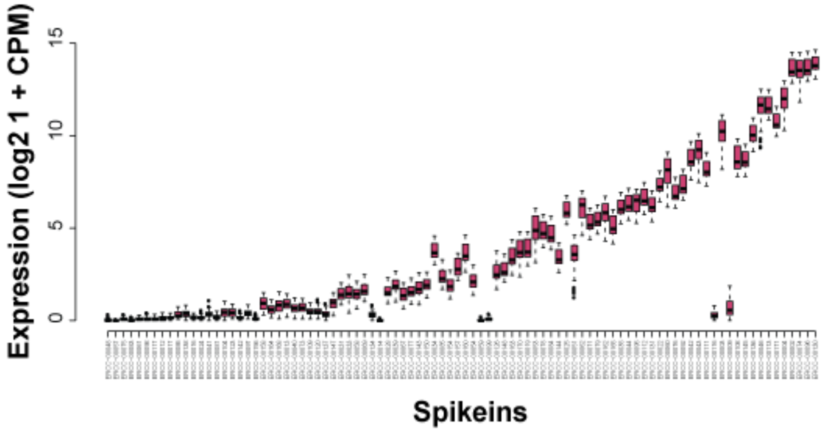

Fig. S5. RNA-sequencing quality control. Consistent expression levels for the spike-ins across the samples. The lower and upper hinges represent the first and third quartiles, respectively. The center bar indicates the median, while the whiskers extend 1.5 times the interquartile range above and below the boxes.

Fig. S6

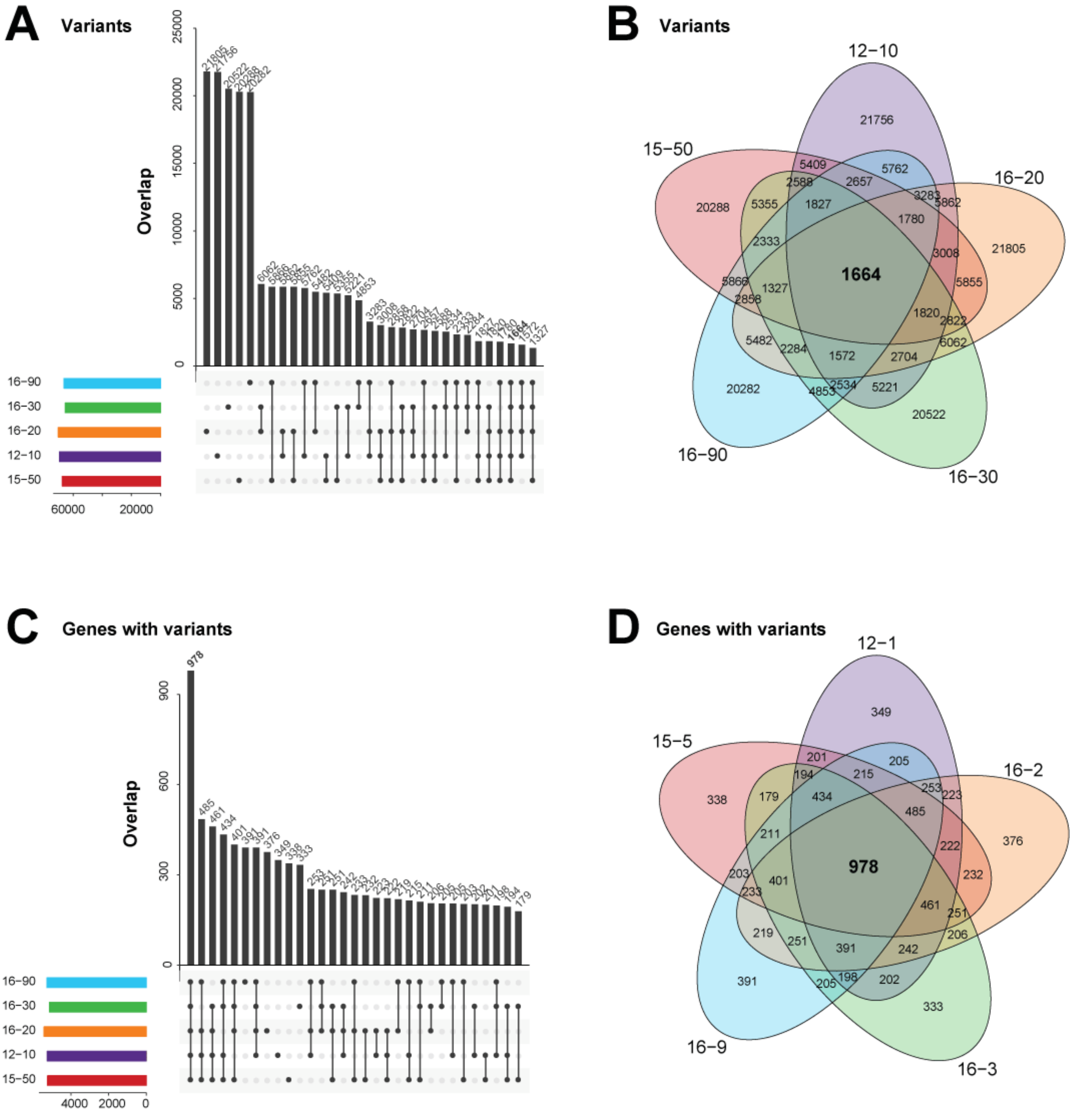

Fig. S6. Genetic similarity across the quads. (A,B) The overlap of these variants called that are in genes are less than 2000 variants (out of a total of 70K per quad). (C,D) The variants fall in common genes (~900 out of ~5000).

Fig. S7

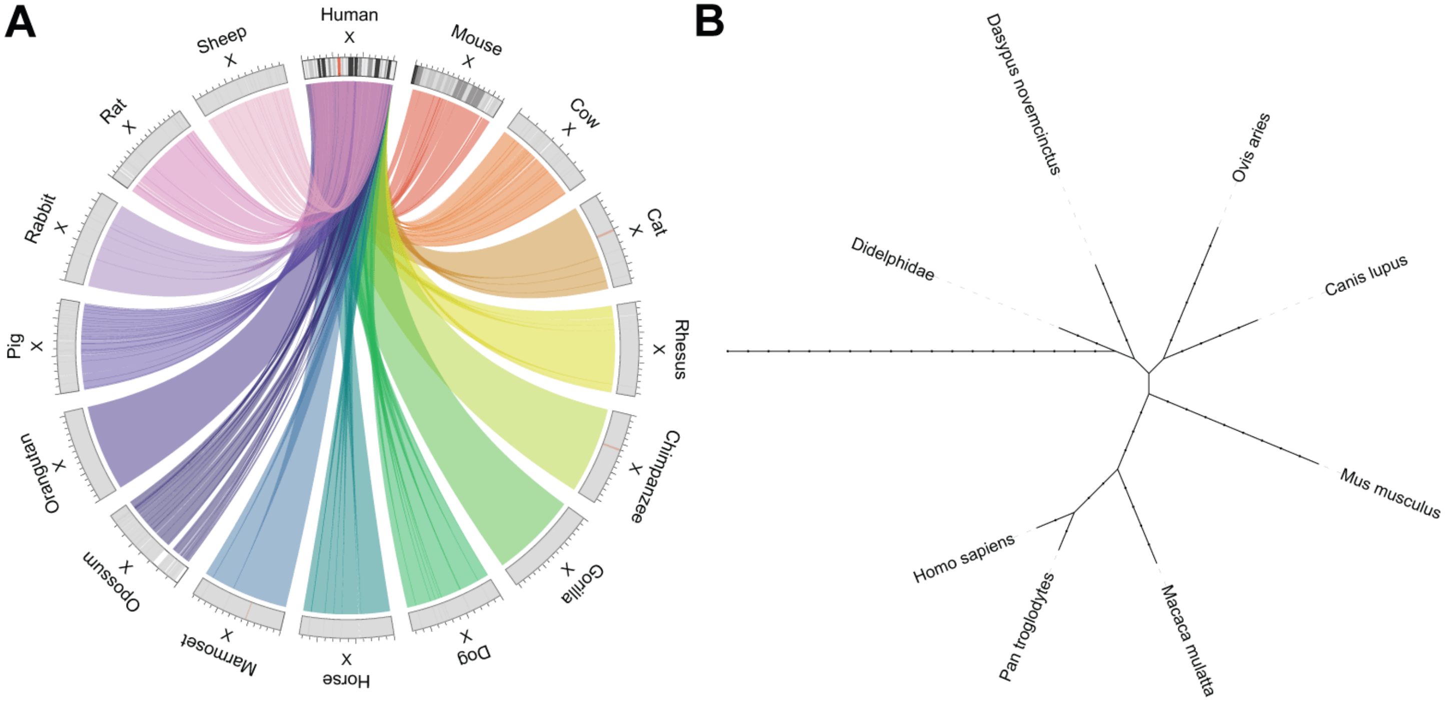

Fig. S7. **X-chromosome synteny.** (A) Circos plot of X-chromosome synteny across annotated species. (B) Phylogenetic comparisons. Plots generated from: http://bioinfo.konkuk.ac.kr/synteny_portal/htdocs/synteny_circos.php and https://phylot.biobyte.de/index.cgi

Fig. S8

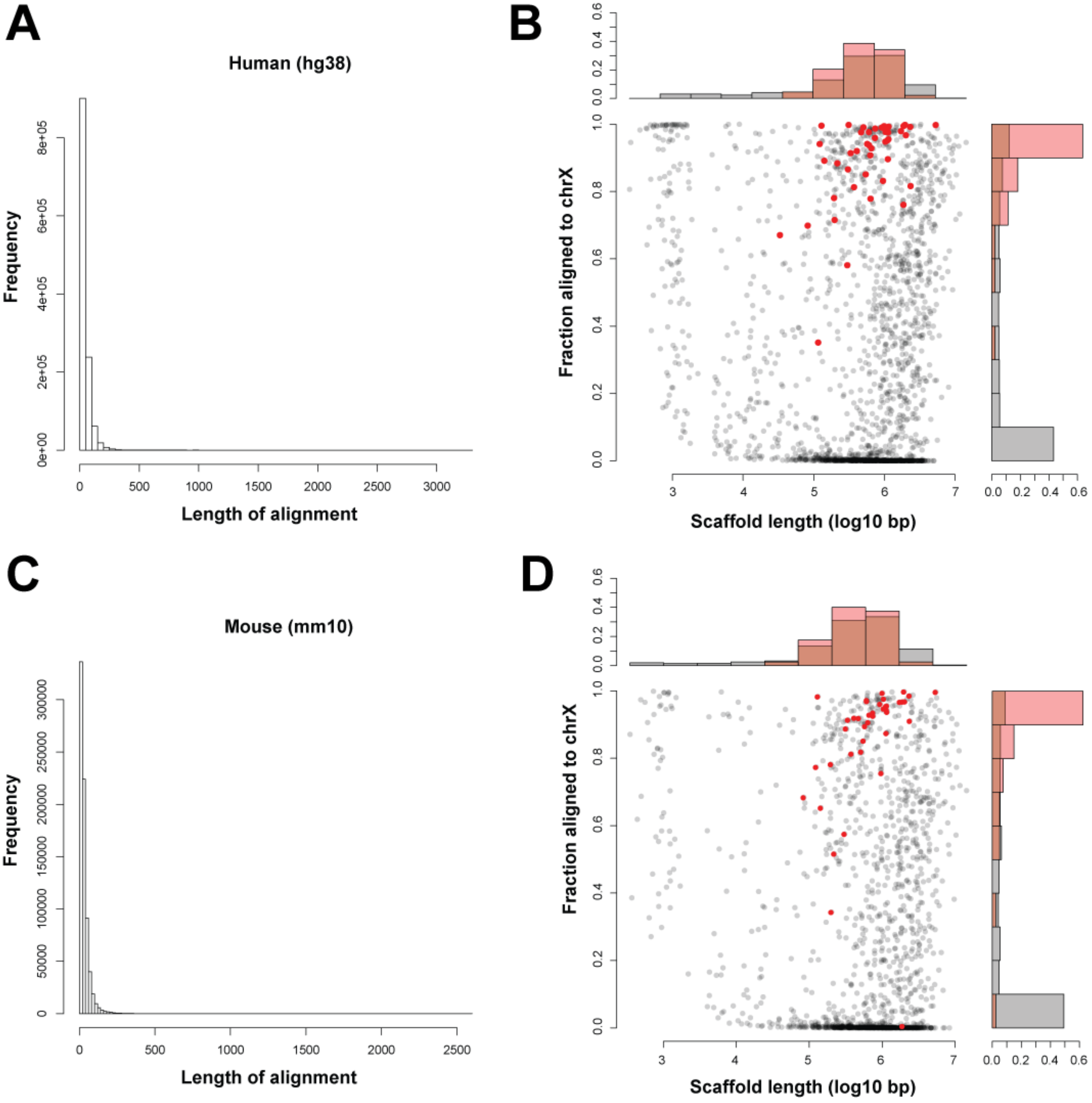

Fig. S8. Scaffold length versus fraction aligned to X-chromosomes. (A) Distribution of alignment lengths of the human X chromosome to armadillo scaffolds. (B) Armadillo scaffold length versus proportion of scaffold that maps to the human X chromosome. (C) and (D) same as above but for mouse. Red points are human and mouse X-chromosome gene homologs (respectively).

Fig. S9

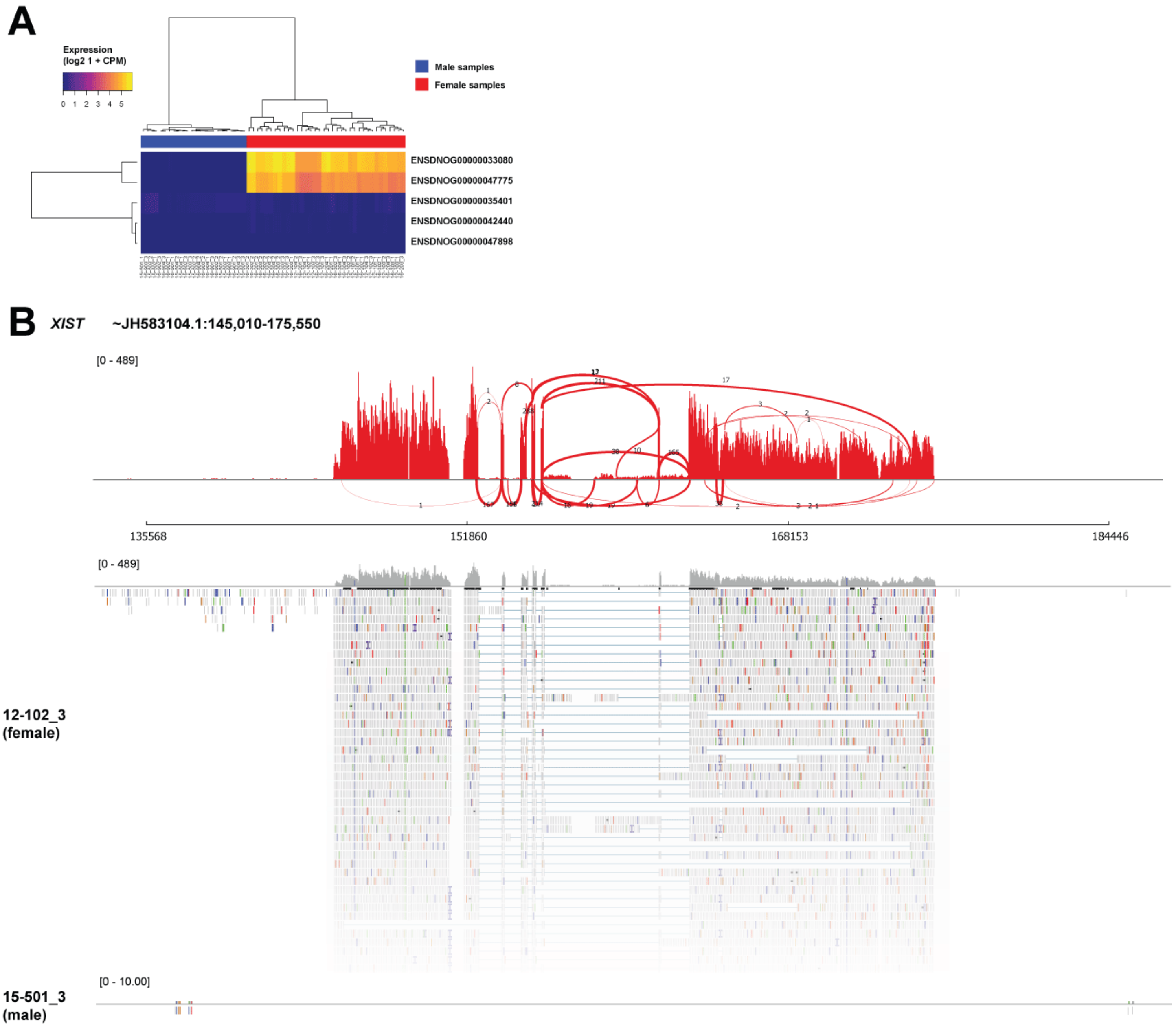

Fig. S9. It eXISTs. (A) Expression of genes annotated near top BLAST hits. (B) Sashimi plot of predicted *XIST*.

Table S1.

Blood collection time points

			Time point 1			Time point 2		
**QuadID**	**Sex**	**Individual ID**	**Collected**	**Received**	**Age**	**Collected**	**Received**	**Age**
12-10	F	12-101	3/1/2017	4/12/2017	5	2/1/2018	3/15/2018	6
12-10	F	12-102	3/1/2017	4/12/2017	5	2/1/2018	3/15/2018	6
12-10	F	12-103	3/1/2017	4/12/2017	5	2/1/2018	3/15/2018	6
12-10	F	12-104	3/1/2017	4/12/2017	5	2/1/2018	3/15/2018	6
15-50	M	15-501	3/1/2017	4/12/2017	2	2/1/2018	3/15/2018	3
15-50	M	15-502	3/1/2017	4/12/2017	2	2/1/2018	3/15/2018	3
15-50	M	15-503	3/1/2017	4/12/2017	2	2/1/2018	3/15/2018	3
15-50	M	15-504	3/1/2017	4/12/2017	2	2/1/2018	3/15/2018	3
16-20	F	16-201	8/1/2017	8/18/2017	1	2/1/2018	3/15/2018	2
16-20	F	16-202	8/1/2017	8/18/2017	1	2/1/2018	3/15/2018	2
16-20	F	16-203	8/1/2017	8/18/2017	1	2/1/2018	3/15/2018	2
16-20	F	16-204	8/1/2017	8/18/2017	1	2/1/2018	3/15/2018	2
16-30	F	16-301	8/1/2017	8/18/2017	1	2/1/2018	3/15/2018	2
16-30	F	16-302	8/1/2017	8/18/2017	1	2/1/2018	3/15/2018	2
16-30	F	16-303	8/1/2017	8/18/2017	1	2/1/2018	3/15/2018	2
16-30	F	16-304	8/1/2017	8/18/2017	1	2/1/2018	3/15/2018	2
16-90	M	16-901	8/1/2017	8/18/2017	1	2/1/2018	3/15/2018	2
16-90	M	16-902	8/1/2017	8/18/2017	1	2/1/2018	3/15/2018	2
16-90	M	16-903	8/1/2017	8/18/2017	1	2/1/2018	3/15/2018	2
16-90	M	16-904	8/1/2017	8/18/2017	1	2/1/2018	3/15/2018	2

			Time point 3		
**QuadID**	**Sex**	**Individual ID**	**Collected**	**Received**	**Age**
12-10	F	12-101	5/10/2018	6/26/2018	6
12-10	F	12-102	5/10/2018	6/26/2018	6
12-10	F	12-103	5/10/2018	6/26/2018	6
12-10	F	12-104	5/10/2018	6/26/2018	6
15-50	M	15-501	5/2/2018	6/26/2018	3
15-50	M	15-502	5/2/2018	6/26/2018	3
15-50	M	15-503	5/2/2018	6/26/2018	3
15-50	M	15-504	5/2/2018	6/26/2018	3
16-20	F	16-201	7/20/2018	9/13/2018	2
16-20	F	16-202	7/20/2018	9/13/2018	2
16-20	F	16-203	7/20/2018	9/13/2018	2
16-20	F	16-204	7/20/2018	9/13/2018	2
16-30	F	16-301	7/20/2018	9/13/2018	2
16-30	F	16-302	7/20/2018	9/13/2018	2
16-30	F	16-303	7/20/2018	9/13/2018	2
16-30	F	16-304	7/20/2018	9/13/2018	2
16-90	M	16-901	8/3/2018	9/13/2018	2
16-90	M	16-902	8/3/2018	9/13/2018	2
16-90	M	16-903	8/3/2018	9/13/2018	2
16-90	M	16-904	8/3/2018	9/13/2018	2

Table S2.
RNA-sequencing summary

**Machine**	NextSeq500
**Sequencing**	PE76
	Dual index
	KAPA polyA

**Flow cell**	HLKF7AF XX	HLJF2AF XX	HHFFKBG X3	HKNNFBG X5	HKNNGBG X5	H7MVWB GX9	H2LFLBG X9
**Library ID**	298826	298827	299555	300489	300490	298975	298976
**Run**	Mid Output	Mid Output	High Output	High Output	High Output	High Output	High Output
**Date**	17-05-09	17-05-09	17-09-15	18-03-29	18-03-29	18-10-19	18-10-19
**Clusters (Raw)**	183029349	201345771	627521395	633345054	597025358	592280827	639878213
**Clusters (PF)**	165467211	179247059	569048477	565141569	545800888	518382260	540853374
**Yield (MBases)**	25151	27246	86495	85902	82962	78794	82210

Table S3.
Mapping rates to the reference genome

SampleID	Total reads	Mapped unique	Fraction mapped	SampleID	Total reads	Mapped unique	Fraction mapped
12-101_1	41602542	36015733	86.57%	15-501_1	35282053	30339942	85.99%
12-101_2	54278544	47385903	87.30%	15-501_2	44446239	38451885	86.51%
12-101_3	40154008	34173405	85.11%	15-501_3	30792841	26110927	84.80%
12-102_1	38973064	32792258	84.14%	15-502_1	37266875	31888980	85.57%
12-102_2	46986854	40564286	86.33%	15-502_2	52735117	46173745	87.56%
12-102_3	34561622	29214558	84.53%	15-502_3	32965973	28177741	85.48%
12-103_1	37362587	32323948	86.51%	15-503_1	40360580	34979716	86.67%
12-103_2	48273632	41533864	86.04%	15-503_2	47881949	41387965	86.44%
12-103_3	37041652	30931023	83.50%	15-503_3	39966441	34243060	85.68%
12-104_1	38406013	33044512	86.04%	15-504_1	41064191	35589149	86.67%
12-104_2	53265459	45975918	86.31%	15-504_2	54408473	47865056	87.97%
12-104_3	39445894	33527681	85.00%	15-504_3	33643439	28342602	84.24%
16-201_1	45474598	39484050	86.83%	16-301_1	38922543	33780878	86.79%
16-201_2	45882806	40261407	87.75%	16-301_2	49669143	43797406	88.18%
16-201_3	34640418	29571671	85.37%	16-301_3	45974587	39159006	85.18%
16-202_1	39279314	34256794	87.21%	16-302_1	39645412	34175041	86.20%
16-202_2	42937510	38040993	88.60%	16-302_2	54415338	48152804	88.49%
16-202_3	37243330	31742796	85.23%	16-302_3	43267015	36715960	84.86%
16-203_1	47244264	41377647	87.58%	16-303_1	41475341	35384624	85.31%
16-203_2	48221483	42381863	87.89%	16-303_2	48007335	42685052	88.91%
16-203_3	42876738	36388842	84.87%	16-303_3	27402266	23012737	83.98%
16-204_1	43357634	37477748	86.44%	16-304_1	42662916	36925309	86.55%
16-204_2	54102026	47703555	88.17%	16-304_2	43762162	38625922	88.26%
16-204_3	41711600	35245490	84.50%	16-304_3	48557116	41159707	84.77%
16-901_1	52162951	43497194	83.39%				
16-901_2	52082789	46009545	88.34%				
16-901_3	43438263	36466131	83.95%				
16-902_1	41859373	35396812	84.56%				
16-902_2	49328126	43342298	87.87%				
16-902_3	35433898	30084997	84.90%				
16-903_1	44266009	37182722	84.00%				
16-903_2	46358744	40791911	87.99%				
16-903_3	43795015	37366506	85.32%				
16-904_1	38380655	32458961	84.57%				
16-904_2	47565012	41494160	87.24%				
16-904_3	33501327	28426115	84.85%				

Table S4.
DNA-sequencing summary from NYGC

**Source Tissue Type**	Blood
**Species**	Dasypus novemcinctus
**Reference Genome**	Other-non_human_DNA
**Library Prep**	TruSeq Nano 450bp
**Coverage/Reads**	30x

Quad	DNA collected from	Sex	Volume (µL)	Concentration (ng/µL)	Mass (ng)	GCN
12-10	12D101_12D102_12D103_12D104	Female	94	17.16	1680.09	7.7
15-50	15F501_15F502_15F503_15F504	Male	94	33.71	3283.28	8.4
16-20	16-201_16-202_16-203_16-204	Female	94	21.45	2099.93	7.8
16-30	16-302_16-303_16-304	Female	41	16.17	1579.78	8.3
16-90	16-901_16-902_16-903_16-904	Male	110	27.38	2836.50	6.0

Table S5.
Mapping rates to personal quadruplet genomes.

SampleID	Total reads	Mapped unique	Fraction mapped	SampleID	Total reads	Mapped unique	Fraction mapped
12-101_1	41602542	34731798	83.48%	15-501_1	35282053	29401615	83.33%
12-101_2	54278544	44553371	82.08%	15-501_2	44446239	36375942	81.84%
12-101_3	40154008	33130726	82.51%	15-501_3	30792841	25377955	82.42%
12-102_1	38973064	31773390	81.53%	15-502_1	37266875	30893161	82.90%
12-102_2	46986854	38314183	81.54%	15-502_2	52735117	43516277	82.52%
12-102_3	34561622	28499980	82.46%	15-502_3	32965973	27524332	83.49%
12-103_1	37362587	31397309	84.03%	15-503_1	40360580	34337702	85.08%
12-103_2	48273632	38664809	80.10%	15-503_2	47881949	39652206	82.81%
12-103_3	37041652	29667242	80.09%	15-503_3	39966441	33282624	83.28%
12-104_1	38406013	31937592	83.16%	15-504_1	41064191	34628513	84.33%
12-104_2	53265459	43318507	81.33%	15-504_2	54408473	44678125	82.12%
12-104_3	39445894	32561207	82.55%	15-504_3	33643439	27535651	81.85%
16-201_1	45474598	38365399	84.37%	16-301_1	38922543	32758685	84.16%
16-201_2	45882806	37553676	81.85%	16-301_2	49669143	41658436	83.87%
16-201_3	34640418	28386059	81.94%	16-301_3	45974587	38107943	82.89%
16-202_1	39279314	33375526	84.97%	16-302_1	39645412	33504750	84.51%
16-202_2	42937510	35557003	82.81%	16-302_2	54415338	46100056	84.72%
16-202_3	37243330	30644433	82.28%	16-302_3	43267015	35661945	82.42%
16-203_1	47244264	40591140	85.92%	16-303_1	41475341	34423491	83.00%
16-203_2	48221483	40302268	83.58%	16-303_2	48007335	40759874	84.90%
16-203_3	42876738	35112101	81.89%	16-303_3	27402266	22311426	81.42%
16-204_1	43357634	36513505	84.21%	16-304_1	42662916	36066795	84.54%
16-204_2	54102026	45112901	83.38%	16-304_2	43762162	36821177	84.14%
16-204_3	41711600	34172098	81.92%	16-304_3	48557116	40042965	82.47%
16-901_1	52162951	42319360	81.13%				
16-901_2	52082789	43831059	84.16%				
16-901_3	43438263	35135909	80.89%				
16-902_1	41859373	34667948	82.82%				
16-902_2	49328126	40601505	82.31%				
16-902_3	35433898	28899493	81.56%				
16-903_1	44266009	36313376	82.03%				
16-903_2	46358744	38302650	82.62%				
16-903_3	43795015	36015299	82.24%				
16-904_1	38380655	31433631	81.90%				
16-904_2	47565012	39127091	82.26%				
16-904_3	33501327	27349728	81.64%				

Table S6.
Reciprocal top BLAST hits for *XIST* in humans

Armadillo ID	Top human hit			
ENSDNOG00000000718	CHR_HSCHR10_1_CTG4:77821512-77822104 [Sequence]	DLG5	593 [Sequence]	86.34 [Alignment]
ENSDNOG00000032486	14:49586580-49586872 [Sequence]	RPS29, AL139099.5, RN7SL1	293 [Sequence]	90.10 [Alignment]
ENSDNOG00000033080	X:73831145-73831260 [Sequence]	XIST, Xist_exon4	116 [Sequence]	95.69 [Alignment]
ENSDNOG00000033288	10:42566677-42567476 [Sequence]	EIF3LP2	801 [Sequence]	89.64 [Alignment]
ENSDNOG00000034036	X:120691217-120691290 [Sequence]		74 [Sequence]	93.24 [Alignment]
	14:74126451-74126520 [Sequence]	LIN52	70 [Sequence]	94.29 [Alignment]
ENSDNOG00000034452	14:49586580-49586879 [Sequence]	RPS29, AL139099.5, RN7SL1	300 [Sequence]	98.33 [Alignment]
ENSDNOG00000035926	X:55741080-55741563 [Sequence]	RRAGB	484 [Sequence]	86.36 [Alignment]
ENSDNOG00000036040	11:5453493-5454420 [Sequence]	HBG2, HBE1, AC087380.1, OR51I2	928 [Sequence]	86.96 [Alignment]
ENSDNOG00000037020	11:5544577-5545088 [Sequence]	HBG2, OR52H1	512 [Sequence]	87.70 [Alignment]
ENSDNOG00000039691	6:134133592-134133852 [Sequence]	RN7SL408P	261 [Sequence]	88.89 [Alignment]
ENSDNOG00000040606	3:11103033-11103275 [Sequence]		243 [Sequence]	72.84 [Alignment]
ENSDNOG00000042044	14:49862649-49862841 [Sequence]	AL627171.2, RN7SL2	193 [Sequence]	93.78 [Alignment]
ENSDNOG00000042440	14:49586588-49586780 [Sequence]	RPS29, AL139099.5, RN7SL1	193 [Sequence]	93.78 [Alignment]
	X:140085873-140086037 [Sequence]	RN7SL727P	165 [Sequence]	89.09 [Alignment]

Armadillo ID	Top human hit			
ENSDNOG00000042960	3:15738515-15738715 [Sequence]	ANKRD28, RN7SL4P	201 [Sequence]	94.53 [Alignment]
ENSDNOG00000045206	16:71137144-71137399 [Sequence]	HYDIN	256 [Sequence]	88.67 [Alignment]
ENSDNOG00000047775	X:73821657-73821724 [Sequence]	TSIX, XIST, XIST_intron	68 [Sequence]	92.65 [Alignment]

Table S7.
Reciprocal top BLAST hits for *XIST* in mouse

Armadillo ID	Top mouse hit			
ENSDNOG00000000718	14:24158165-24158756 [Sequence]	Dlg5	592 [Sequence]	79.90 [Alignment]
ENSDNOG00000032486	12:69159295-69159587 [Sequence]	Rn7s1, AC099934.2, AC099934.1	293 [Sequence]	89.42 [Alignment]
ENSDNOG00000033080	X:103469677-103469758 [Sequence]	Gm26992, Tsix, Xist, Gm27927	82 [Sequence]	93.90 [Alignment]
ENSDNOG00000033288	15:79089463-79089960 [Sequence]	Eif3l	498 [Sequence]	88.96 [Alignment]
ENSDNOG00000034036	18:80197662-80197795 [Sequence]	Rbfa	134 [Sequence]	85.82 [Alignment]
	X:155157596-155157655 [Sequence]		60 [Sequence]	91.67 [Alignment]
ENSDNOG00000034452	12:69159295-69159594 [Sequence]	Rn7s1, AC099934.2, AC099934.1	300 [Sequence]	98.33 [Alignment]
ENSDNOG00000035926	14:24164394-24164579 [Sequence]	Dlg5	186 [Sequence]	89.25 [Alignment]
ENSDNOG00000036040	7:104049443-104050316 [Sequence]	Olfr643, Olfr642	875 [Sequence]	85.60 [Alignment]
ENSDNOG00000037020	7:104049443-104050316 [Sequence]	Olfr643, Olfr642	875 [Sequence]	85.60 [Alignment]
ENSDNOG00000039691	12:69159400-69159583 [Sequence]	Rn7s1, AC099934.2, AC099934.1	184 [Sequence]	96.20 [Alignment]
ENSDNOG00000040606	12:63684161-63684362 [Sequence]		202 [Sequence]	72.77 [Alignment]
ENSDNOG00000042044	6:69516344-69516539 [Sequence]	Rn7s6, AC156953.1	196 [Sequence]	92.86 [Alignment]
ENSDNOG00000042440	12:69159314-69159495 [Sequence]	Rn7s1, AC099934.2	182 [Sequence]	94.51 [Alignment]
	X:87899315-87899489 [Sequence]	Il1rapl1, Gm24812	175 [Sequence]	84.00 [Alignment]
ENSDNOG00000042960	6:69516339-69516539 [Sequence]	Rn7s6, AC156953.1	201 [Sequence]	94.03 [Alignment]

Armadillo ID	Top mouse hit			
ENSDNOG00000045206	6:131253488-131253758 [Sequence]	Gm5582	276 [Sequence]	80.43 [Alignment]
ENSDNOG00000047775	X:103461220-103461264 [Sequence]	Gm26992, Tsix, Xist, Gm27733	45 [Sequence]	88.89 [Alignment]
