# Supplementary material for: The transcriptional legacy of developmental stochasticity
Source: Nat Commun. 2023 Nov 9;14:7226. doi: 10.1038/s41467-023-43024-5 (PMC10632366; doi:10.1038/s41467-023-43024-5)
Supplement: Supplementary file 3 — Reporting Summary [file 41467_2023_43024_MOESM3_ESM.pdf]

nature portfolio

Corresponding author(s): Jesse Gillis

Last updated by author(s): Oct 18, 2023

Reporting Summary

Nature Portfolio wishes to improve the reproducibility of the work that we publish. This form provides structure for consistency and transparency in reporting. For further information on Nature Portfolio policies, see our Editorial Policies and the Editorial Policy Checklist.

Statistics

For all statistical analyses, confirm that the following items are present in the figure legend, table legend, main text, or Methods section.

n/a	Confirmed	
☐	☒	The exact sample size (n) for each experimental group/condition, given as a discrete number and unit of measurement
☐	☒	A statement on whether measurements were taken from distinct samples or whether the same sample was measured repeatedly
☐	☒	The statistical test(s) used AND whether they are one- or two-sided<br>*Only common tests should be described solely by name; describe more complex techniques in the Methods section.*
☒	☐	A description of all covariates tested
☐	☒	A description of any assumptions or corrections, such as tests of normality and adjustment for multiple comparisons
☐	☒	A full description of the statistical parameters including central tendency (e.g. means) or other basic estimates (e.g. regression coefficient) AND variation (e.g. standard deviation) or associated estimates of uncertainty (e.g. confidence intervals)
☐	☒	For null hypothesis testing, the test statistic (e.g. F, t, r) with confidence intervals, effect sizes, degrees of freedom and P value noted<br>*Give P values as exact values whenever suitable.*
☒	☐	For Bayesian analysis, information on the choice of priors and Markov chain Monte Carlo settings
☒	☐	For hierarchical and complex designs, identification of the appropriate level for tests and full reporting of outcomes
☐	☒	Estimates of effect sizes (e.g. Cohen's d, Pearson's r), indicating how they were calculated

Our web collection on statistics for biologists contains articles on many of the points above.

Software and code

Policy information about availability of computer code

Data collection	We mapped DNA-sequencing reads using BWA and called variants using the GATK Unified Genotyper v3.6.0. We generated personalized genomes using g2gtools v0.2.0 and indexed them using samtools v1.9.RNA-sequencing reads were mapped using STAR v2.7 and were quality controled using GATK v3.6.0 and IGVTools v2.3.80.
Data analysis	We identified X chromosome scaffolds using the UCSC chain/liftover files and BLAST. All other analyses are based on R custom code found at https://github.com/sarbal/ayotochtli and archived at https://doi.org/10.5281/zenodo.8433151.

For manuscripts utilizing custom algorithms or software that are central to the research but not yet described in published literature, software must be made available to editors and reviewers. We strongly encourage code deposition in a community repository (e.g. GitHub). See the Nature Portfolio guidelines for submitting code & software for further information.

Data

Policy information about availability of data

All manuscripts must include a data availability statement. This statement should provide the following information, where applicable:

- Accession codes, unique identifiers, or web links for publicly available datasets
- A description of any restrictions on data availability
- For clinical datasets or third party data, please ensure that the statement adheres to our policy

All data are available in the main text, source data, or the supplementary materials. The accession numbers for the sequencing datasets reported in this paper have been deposited in Gene Expression Omnibus (GEO) under GSE141951 and Sequence Read Archive (SRA) under SRP233269.

Field-specific reporting

Please select the one below that is the best fit for your research. If you are not sure, read the appropriate sections before making your selection.

☒ Life sciences ☐ Behavioural & social sciences ☐ Ecological, evolutionary & environmental sciences

For a reference copy of the document with all sections, see nature.com/documents/nr-reporting-summary-flat.pdf

Life sciences study design

All studies must disclose on these points even when the disclosure is negative.

Sample size	Our study profiled five quadruplets of armadillos (20 armadillos in total), with genome-wide measurements at 3 time points for each individual (60 total samples). This was a standard sample of convenience that was able to detect canalized allelic signatures. Specifically, the study included 3 unrelated female quadruplets, which were sufficient to observe highly variable patterns of X-inactivation skews. Together, the 5 quadruplets contributed SNPs that spanned several thousands of genes, enabling us to reliably detect early determination of allelic imbalance. As detailed in the main text, we performed post-hoc power analyses to assess the number of quadruplets and read coverage needed to detect allelic imbalances in the autosomes. In these analyses, we empirically simulated allele-specific expression for random SNPs (n=100 independent simulations). We found that for our read coverage and sample size, we are powered to detect autosomal epigenetic events arising as late as the 10,000 cell stage.
Data exclusions	No data were excluded from the analyses.
Replication	Results were replicated over five sets of genetically unrelated quadruplets and three of these sets were used for female-specific analyses.
Randomization	The study design did not involve multiple experimental groups.
Blinding	Blinding was not relevant to study design (only one experimental group).

Reporting for specific materials, systems and methods

We require information from authors about some types of materials, experimental systems and methods used in many studies. Here, indicate whether each material, system or method listed is relevant to your study. If you are not sure if a list item applies to your research, read the appropriate section before selecting a response.

Materials & experimental systems

n/a	Involved in the study	
☒	☐	Antibodies
☒	☐	Eukaryotic cell lines
☒	☐	Palaeontology and archaeology
☐	☒	Animals and other organisms
☒	☐	Human research participants
☒	☐	Clinical data
☒	☐	Dual use research of concern

Methods

n/a	Involved in the study	
☒	☐	ChIP-seq
☒	☐	Flow cytometry
☒	☐	MRI-based neuroimaging

Animals and other organisms

Policy information about studies involving animals; ARRIVE guidelines recommended for reporting animal research

Laboratory animals	The study did not involve laboratory animals.
Wild animals	Pregnant females were captured using long-handled nets at night from the wild in 2012, 2015 and 2016. Capture of the pregnant armadillos was done during the spring to avoid collecting females who were nursing young, but were potentially pregnant. The animals were retrieved from the nets and placed in kennels for immediate transport to the holding facility at the University of the Ozarks, Clarksville, AR.
Field-collected samples	The pregnant females were kept in outdoor pens that had burrows where they gave birth to the quadruplets. The babies were kept with the mothers until they were observed foraging at about 6-10 weeks postnatal age. After separation from the mothers, the animals were housed together in semi-outdoors pens (rubber covered concrete floor under a roof). Most litters in the semi-outdoor pens shared the pen with another litter (2 litters per pen). All adults and young over 49 days postnatal age (pna) were fed a mix of dry dog and cat chicken-and-rice chow moistened with water—in an approximate ratio by volume of 1:1:2. Adults were provided 0.75-1.5 cups (indoor-outdoor) of moistened chow once a day during the gestation and 1.25-1.75 cups per day during known or suspected lactation. Animals housed in outdoor enclosures were able to forage as well. Occasionally, a raw egg and earthworms were provided in addition to the chow. Litters were fed replacement formula of reconstituted Esbilac puppy replacement formula until old enough. After 35 days pna, the diet was gradually transitioned to that of adult by 49-56 days pna. The wild-caught females were

administered 0.15 ml Ivermectin SC, and Exceed antibiotic if they showed any wounds or abscesses. Adults were dewormed every 6-8 weeks with Panacure on chow for three consecutive days, or with 0.2 ml Ivermectin on food. The babies were treated once with Panacure on chow for three consecutive days.
At four to five months of age, the animals were delivered to the National Hansen's Disease Program (NHDP) facility in Baton Rouge, LA where they were placed in pairs in modified rabbit cages. They were fed the same dry food as that given at the Arkansas facility. After a period of adaption of approximately one year, the animals were treated with Penicillin (1.0 mL) and dewormed with Ivermectin (0.1 mL) and Praziquantel (0.4 mL). Prednisone (10mg/mL) was also given at this time.
The pilot study consisted of two sets of quadruplets (12-10 and 15-50), and then later blood was obtained from quadruplets 16-20, 16-30 and 16-90. During the course of the year, the original two of the five sets of armadillo quadruplets were infected intravenously in the saphenous vein with 1x109 Mycobacterium leprae derived from athymic nude mice – both after the first time point was collected. Blood was collected at different time points throughout the course of disease and at 18-24 months post-infection, the animals were humanely sacrificed when they developed heavy M. leprae dissemination with severe hypochromic microcytic anemia. Animals were euthanized by exsanguination under anesthesia/analgesia at double the normal doses. Exsanguinated animals are wrapped in plastic and deposited at 4C to assure non-recovery.

Ethics oversight

The study protocol was approved by the NHDP Institutional Animal Care and Use Committee.

Note that full information on the approval of the study protocol must also be provided in the manuscript.
